# Dough rheology, antioxidants, textural, physicochemical characteristics, and sensory quality of pizza base enriched with onion (*Allium cepa* L.) skin powder

**DOI:** 10.1038/s41598-020-75793-0

**Published:** 2020-10-29

**Authors:** Narashans Alok Sagar, Sunil Pareek

**Affiliations:** Department of Agriculture and Environmental Sciences, National Institute of Food Technology Entrepreneurship and Management, Plot No. 97, Sector 56, HSIIDC Industrial Estate, Kundli, Sonipat, Haryana 131028 India

**Keywords:** Biochemistry, Biological techniques

## Abstract

In the present research, wheat flour was replaced with onion skin powder (OSP) in 2%, 3.5%, and 5% concentration along with control to produce different pizza base variants. Prepared pizza doughs and base were investigated for different quality parameters. Rheology revealed that increased concentration of OSP elevated the storage modulus (G’) (solid nature) of pizza doughs. Colour measurement of both the doughs and pizza base exhibited lightness in control (L* 86.46 ± 0.39) and darkness in 5% OSP variant (L* 46.43 ± 0.69). Physicochemical investigation showed no significant difference however, a gradual increase was obtained in fiber, water, and oil holding capacity of pizza base. Texture properties showed that the addition of OSP imparted an increased trend of hardness i.e. 5% OSP variant had maximum hardness (14.87 ± 0.20 N). A higher level of total phenols, total flavonoids, and antioxidant activity was obtained in fortified products, which exhibits onion skin as a natural source of antioxidants for functional foods. Sensory evaluation revealed OSP 2% as the most accepted variant in terms of overall acceptability. The storage study of the pizza base revealed that controlled environment was the best-suited atmosphere for a longer shelf-life of pizza base.

## Introduction

Now-a-days, the nutrition and health aspects of food products have become important parameters for consumers. As a result, demand for functional and innovative foods has been enhanced. Functional foods are prepared by the fortification technique in which bioactive compounds are incorporated into food products^1^. Antioxidants and fiber-rich food products are the important categories of functional foods which comprise properties to mitigate various health disorders^2,3^. According to the scientists, deficiency of antioxidants in daily food is the main reason for reactive oxygen species (ROS) in the body which consequently leads to various health diseases. There is a need to search a suitable natural source of antioxidants which could neutralize the excess ROS concentration in the human body^4^.

Huge onion waste is produced during postharvest operations and processing of onion products. Onion skin comprises approximately 60% of total waste which contains a significantly higher content of flavonoids, phenols, and antioxidants along with other bioactive compounds than other horticultural commodities^5,6^. Furthermore, onion and its by-products showed several in vitro and in vivo biological activities such as antioxidant activity, anti-microbial properties, antifungal, anti-inflammatory, and anti-carcinogenic property^7,8^. It can be stated that the utilization of onion skin as a potential ingredient of functional food may provide two things i.e. natural antioxidant-rich food and a solution for environmental pollution^9^.

Bakery items are considered as the best products for fortification because 70% daily calorie requirement is contributed by bread^10^. There are many studies available on antioxidant-fortified bread^5,11,12^ which confirmed a higher level of available antioxidants in enriched products, however, the effect of onion skin addition on the properties of pizza dough and base has not been sufficiently studied^13^. In contrast to bread, pizza possesses higher crust area than crumb because pizza dough is generally prepared with lower amount of oil and water (though there is no standard recipe) and pressed into flat shape^14^ and therefore, it is being consumed and liked by group of people worldwide^15^. Thus, both products may have differences in textural properties and shelf-life which make the pizza base, a suitable product for the present study.

The scientific studies on pizza base are scanty, therefore, the present experiment was carried out with the objective to find out the effect of addition of onion skin powder (OSP) on the rheological behaviour of pizza dough. After baking, organoleptic parameters and textural properties were investigated to find the effect of OSP enrichment. Moreover, total phenols and antioxidants were also analyzed in whole pizza base after OSP addition^16^. As important parameters, color analysis, physicochemical parameters, and shelf-life of the pizza base were also determined.

## Results

### Rheological properties of dough

**Figure 1:**
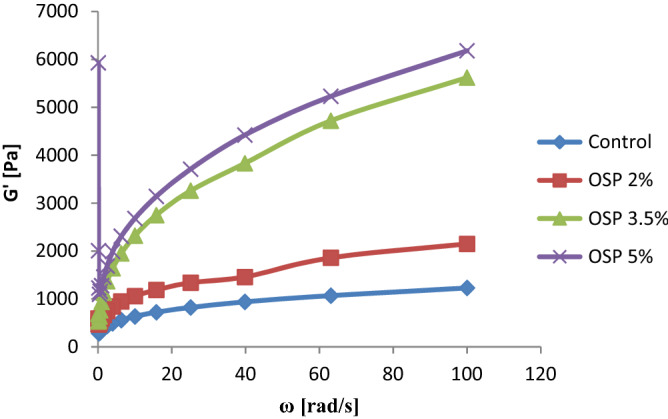
Storage modulus (G’) vs. angular frequency (ω) of pizza dough fortified with OSP in different concentrations.

Analysis of all results showed that the storage modulus (G’) was better than the viscous modulus (G’’) to represent rheological behavior of dough during fermentation and baking and G’ exhibited a higher correlation with pizza properties. As per the graph, G’ (Fig. 1) and G’’ (not shown) were enhanced with angular frequency (ω) in enriched samples, corresponding a predominant solid nature of the doughs (G’ > G’’). Samples were compared by G’ value (within linear range) at ω = 10 rad/s. The dough strength (G’) increased in following order: control < OSP 2% < OSP 3.5% < 5% OSP (Fig. 1). It revealed that addition of OSP in dough significantly increased the G’ value of the pizza dough because of its fiber content and flavonoids such as quercetin, kaempferol, and luteolin that changed the sample matrix to more solid nature of enriched doughs compare to control. A significant (P < 0.05) difference was noted between all the variants of pizza dough.

### Analysis of color attributes of pizza dough and base

The color values of pizza dough and base were measured on L*, a*, b* color scale, and results are given in Table 1. A significant difference (P < 0.05) was observed in the color parameters of pizza dough and base variants. Pizza dough values showed a significant decrease in lightness with the increased concentration of OSP. As a result, control dough exhibited maximum lightness (86.46 ± 0.39), and OSP 5% dough sample had the least lightness (46.43 ± 0.69). Similarly for a* value, control dough exhibited more greenness (− 1.05 ± 0.02) than enriched samples i.e. OSP 2% dough (6.54 ± 0.02) and OSP 3.5% (9.85 ± 0.36) while OSP 5% dough showed maximum redness (11.50 ± 0.29). Moreover, a gradual decrease was obtained in b* parameter which represents blueness (−) or yellowness ( +). Control dough had highest b* value (17.26 ± 0.25) i.e. maximum blueness and dough with 5% OSP showed the lowest b* value (7.70 ± 0.02) i.e. maximum yellowness. In case of pizza base, a similar trend was observed in color parameters as recorded in pizza dough. The only difference was the shifting of the value to lower side. The control base showed maximum lightness (70.27 ± 1.15), minimum redness (7.59 ± 0.92), and maximum blueness (31.17 ± 1.16) whereas OSP 5% base exhibited darkest color (43.46 ± 1.04), maximum redness (9.55 ± 0.24), and highest yellowness (12.13 ± 0.61) (Table 1). There was a significant difference (P < 0.05) among all pizza base variants.

**Table 1:** Color values of fortified pizza dough and base with OSP.

Sample	Pizza dough				Pizza base			
	L*	a*	b*	*∆E*	L*	a*	b*	*∆E*
Control	86.46 ± 0.39^d^	− 1.05 ± 0.02^a^	17.26 ± 0.25^d^	82.96 ± 0.40^d^	70.27 ± 1.15^d^	7.59 ± 0.92^a^	31.17 ± 1.16^d^	37.15 ± 1.18^a^
OSP 2%	57.43 ± 0.34^c^	6.54 ± 0.02^b^	13.29 ± 0.24^c^	54.93 ± 0.23^c^	55.31 ± 0.95^c^	9.07 ± 0.27^b^	20.35 ± 0.15^c^	43.92 ± 1.66^b^
OSP 3.5%	49.26 ± 0.15^b^	9.85 ± 0.36^c^	9.84 ± 0.12^b^	47.49 ± 0.87^b^	48.40 ± 0.52^b^	10.23 ± 0.08^c^	15.85 ± 0.83^b^	49.54 ± 0.47^c^
OSP 5%	46.43 ± 0.69^a^	11.50 ± 0.29^d^	7.70 ± 0.02^a^	43.98 ± 0.60^a^	43.46 ± 1.04^a^	9.55 ± 0.24^bc^	12.13 ± 0.61^a^	52.99 ± 0.93^d^

Means with different letters (a-d) in the same column are significantly different (P < 0.05) by Duncan’s multiple range test.

### Textural properties of pizza base

Textural properties are also referred as mechanical attributes. The baked pizza base variants were compressed twice between automated plates of texture analyzer which imitates jaw action. Statistical analysis revealed that the addition of OSP significantly affected all the textural parameters of pizza base (Table 2). Hardness and chewiness increased in following order: control < OSP 2% < OSP 3.5% < OSP 5%. It showed that higher OSP concentrations lead to the hardness because of its fiber content. Whereas no significant change (P < 0.05) was detected in springiness of the samples by OSP addition. Cohesiveness showed following decreasing trend in samples: OSP 5% > control > OSP 2% > OSP 3.5%. Resilience was also increased gradually but no significant difference (P < 0.05) was reported between samples. Control base had 0.49 ± 0.07 resilience and OSP 5% base showed 0.90 ± 0.01 resilience.

**Table 2:** Effect of OSP enrichment on the textural profile of pizza base variants.

Sample	Hardness (N)	Springiness	Chewiness (N)	Cohesiveness	Resilience
Control	8.54 ± 0.50^a^	0.93 ± 0.01^a^	6.23 ± 0.25^a^	0.84 ± 0.01^c^	0.49 ± 0.07^a^
OSP 2%	10.12 ± 0.21^b^	1.01 ± 0.02^b^	9.88 ± 0.38^b^	0.71 ± 0.03^b^	0.70 ± 0.02^b^
OSP 3.5%	11.44 ± 0.51^b^	0.99 ± 0.04^a^	11.22 ± 0.46^b^	0.67 ± 0.02^a^	0.85 ± 0.01^c^
OSP 5%	14.87 ± 0.20^c^	1.00 ± 0.03^b^	16.22 ± 0.18^c^	0.90 ± 0.02^d^	0.90 ± 0.01^c^

Means with common letters in the same column are not significantly different (P < 0.05) by Duncan’s multiple range test.

### Physicochemical properties of pizza base

The effect of OSP addition in pizza base was also investigated for physicochemical analysis. It was observed that no significant change occurred in total fat and moisture content of pizza base after the addition of OSP probably due to the fiber and ash content of the OSP. A non-significant change was obtained in moisture contents of the variants. A slight change was reported in crude protein, crude fiber, and carbohydrates because of the available fiber and carbohydrate content of the OSP. Crude protein and crude fiber content was increased with the successive increment of the OSP ratio in pizza base (Table 3). There was a significant difference (P < 0.05) between the samples. Additionally, OSP addition showed a non-significant gradual decrease in the concentration of carbohydrates of base samples. Carbohydrate content was successively decreased with the increase in OSP content in samples (Table 3).

**Table 3:** Physicochemical properties of pizza base variants fortified with OSP.

Sample	Moisture content (%)	Crude protein (%)	Crude fiber (%)	Carbohydrates (%)	Total fat (%)	Crude ash (%)	WHC	OHC
Control	13.88 ± 0.62^a^	10.98 ± 0.02^a^	0.43 ± 0.01^a^	62.96 ± 0.76^b^	10.67 ± 0.16^a^	1.08 ± 0.02^a^	6.32 ± 0.11^a^	4.22 ± 0.04^a^
OSP 2%	14.19 ± 0.74^a^	11.09 ± 0.02^b^	0.49 ± 0.02^b^	62.11 ± 0.70^b^	10.88 ± 0.04^b^	1.23 ± 0.02^b^	7.58 ± 0.07^b^	5.14 ± 0.07^b^
OSP 3.5%	15.03 ± 1.72^ab^	11.19 ± 0.01^c^	0.77 ± 0.02^c^	59.09 ± 1.73^a^	10.93 ± 0.04^b^	1.92 ± 0.03^c^	8.39 ± 0.06^c^	6.15 ± 0.14^c^
OSP 5%	16.48 ± 0.12^c^	11.27 ± 0.01^d^	0.98 ± 0.04^d^	58.70 ± 0.19^a^	10.98 ± 0.02^b^	2.69 ± 0.07^d^	8.99 ± 0.18^d^	7.22 ± 0.12^d^

Means with common letters in the same column are not significantly different (P < 0.05) by Duncan’s multiple range test.

*WHC* water holding capacity, *OHC* oil holding capacity expressed as g of water/oil held by g of sample.

Apart from this, effect of OSP addition on water holding capacity (WHC) and oil holding capacity (OHC) was also investigated. Results revealed that WHC and OHC of base samples were enhanced gradually by the addition of OSP and a significant difference (P < 0.05) was observed between samples. WHC increased in following order: control < 2% OSP < 3.5% OSP < 5% OSP of pizza base.

### Total phenolic content and total flavonoid content of product extracts

**Figure 2:**
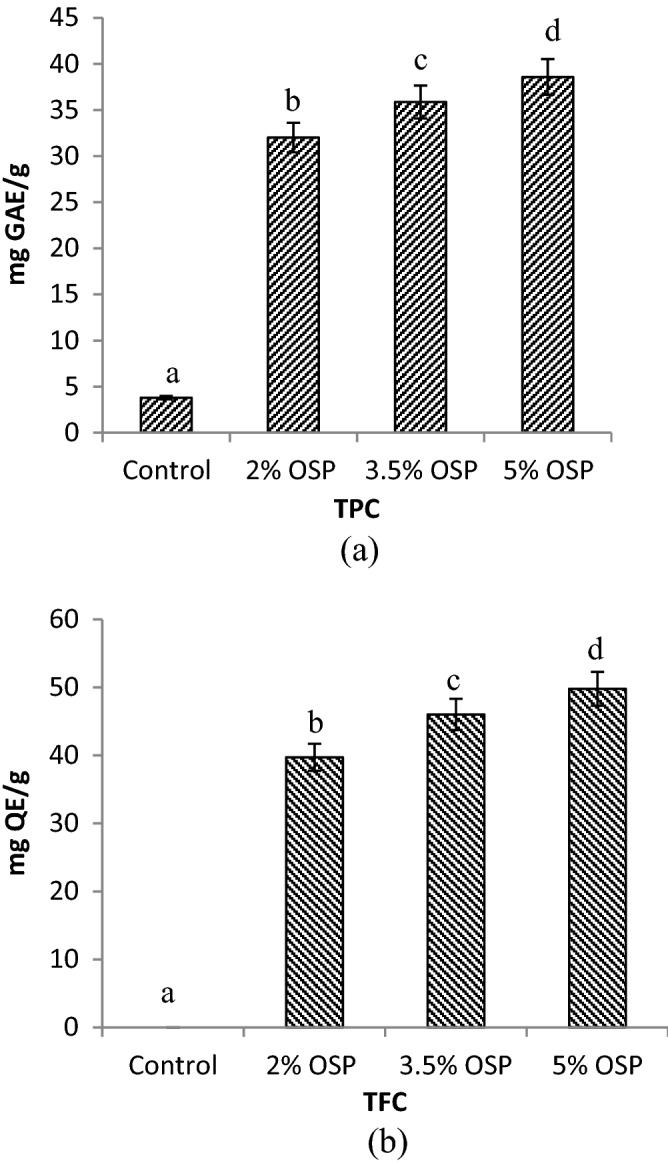
Total phenolic content (TPC) **(a)** and total flavonoid content (TFC) **(b)** of pizza enriched with OSP. Columns with different superscript letters in the same figure are significantly different (P < 0.05).

It was found that TPC and TFC were increased significantly in the final products after the addition of OSP (Fig. 2) because of onion skin of cv. ‘NHRDF Red’ had a maximum amount of quercetin and total phenols^16^. In TPC analysis, OSP 2% and OSP 3.5% variants contained respectively 8-times and 9-times higher total phenols than control while the base with 5% OSP possessed maximum concentration of TPC (Fig. 2a). The variants of pizza base were significantly (P < 0.05) different from each other.

In the case of TFC, a similar trend of increment was reported in fortified base variants. Total flavonoid content was found absent in control sample while a significant (P < 0.05) increase was observed in TFC with respect to the addition of OSP content in the samples. Samples OSP 2%, OSP 3.5% and OSP 5% had approximately 40-times, 46-times and 50-times increase in TFC, respectively than control in mg QE/g DW concentration (Fig. 2b).

### Antioxidant activity of product extracts

**Figure 3:**
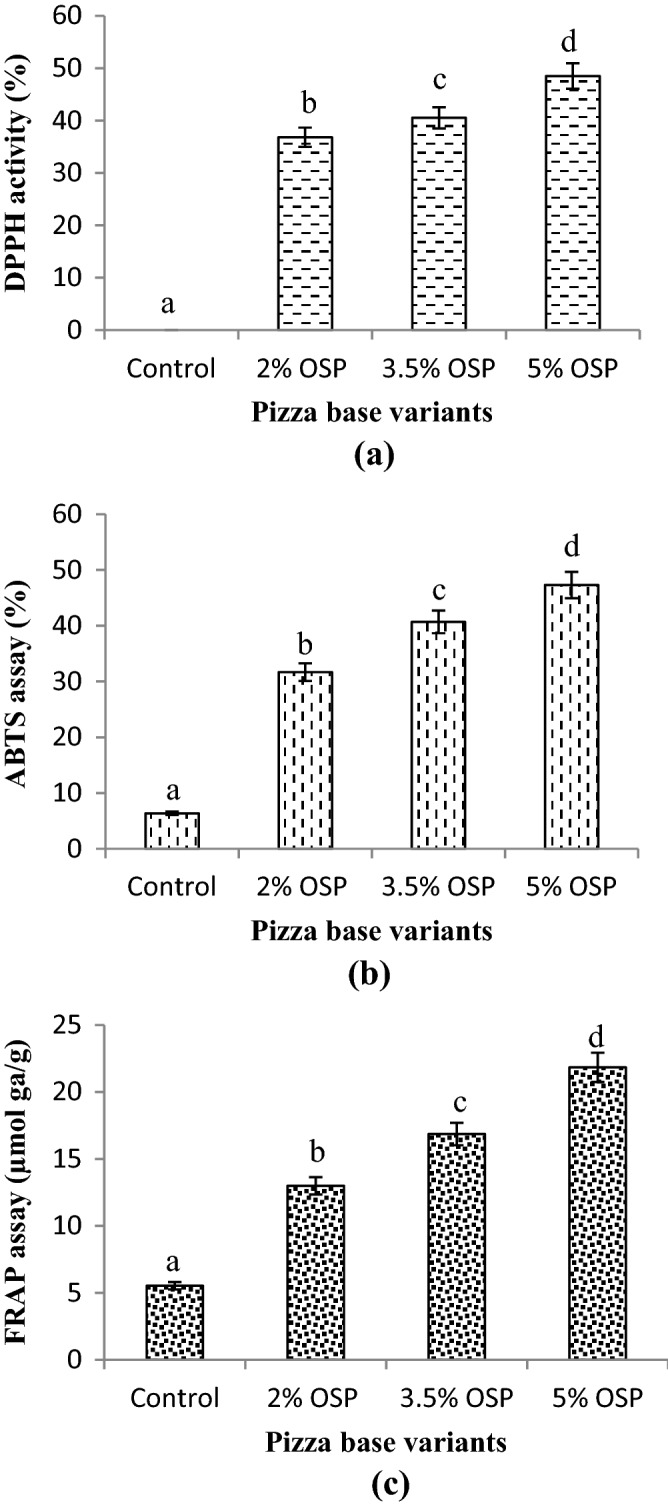
Antioxidant activity of pizza base variants enriched with onion skin powder (OSP): **(a)** DPPH antioxidant activity, **(b)** ABTS radical activity, **(c)** FRAP antioxidant assay. Columns with different superscript letters in the same figure are significantly different (P < 0.05).

The antioxidant activity of fortified products was investigated by three different methods i.e. DPPH antioxidant assay (Fig. 3a), ABTS assay (Fig. 3b), and FRAP assay (Fig. 3c). All the assays showed increased antioxidant activity in final products with a progressive increase in OSP addition from 2 to 5%. The pizza variant with 5% OSP showed maximum antioxidant activity in all the tests whereas the lowest antioxidant activity was found in control variant. For instance, OSP 5% base showed approximately 50-times, 8-times, and 4-times higher antioxidant activity for DPPH, ABTS, and FRAP over control. The respective range of ABTS and FRAP assay was recorded between 6.35 and 47.30% and 5.53–21.84 µmol gallic acid/g in which the lowest value represented control while higher indicated variant with 5% OSP. This significant (P < 0.05) increase occurred in the values due to a higher amount of phenolics (289.04 ± 1.31 mg GAE/g DW) and antioxidant potential (94.17 ± 0.20%) of utilized OSP^16^.

### Sensory analysis of pizza base

**Figure 4:**
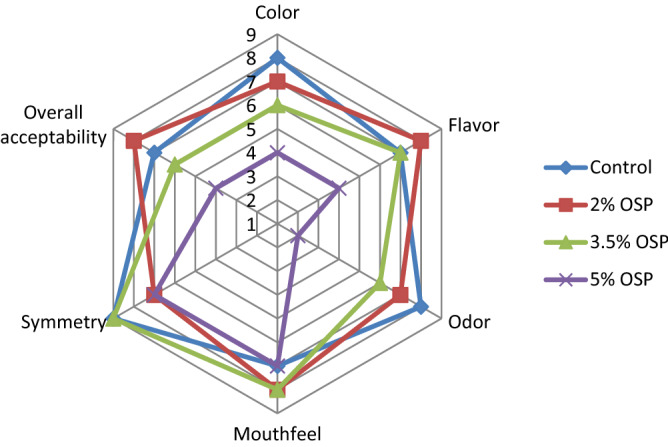
Sensory evaluation of the pizza base variants.

OSP addition imparted darker color to fortified variants of the pizza base. The analysis revealed that mouthfeel was best accepted for OSP 2% and OSP 3.5% mixed pizza base. However, 5% OSP content imparted unpleasant flavor and odor (aroma) (Fig. 4). The darker color of the OSP 5% variant over control might have a negative impact on the consumers which leads to the unacceptability of the product. Variants with 3.5% OSP and 5% OSP showed a significant (P < 0.05) decrease in overall acceptability. In terms of flavor, mouthfeel, and overall acceptability, the variant with partial replacement (2%) of OSP in the pizza base was preferred. However, OSP 5% variant was rated comparatively lower because of higher content of OSP which might lead to unacceptable color, pungent odor and undesirable mouthfeel but consumers who prefer pungent odor and rich flavor may accept the OSP 5% variant of pizza base (Fig. 4).

### Shelf-life study of the pizza base

Comparative microbial study of all the samples was done in two storage conditions: ambient (28 ± 2 ℃) and refrigerated (5 ± 2 ℃). In ambient storage condition, initially, no bacterial and fungal growth was observed but on 3rd day, control had higher bacterial (1.5 × 10^4^ cfu/g) and fungal counts (2.5 × 10^4^ cfu/g) than fortified variants (Fig. S1). Further, a gradual increase was recorded in microbial load up to 7th day of storage in ambient condition (Table 4). It was observed that the control variant had a higher microbial count than fortified samples because OSP worked as a natural preservative due to its flavonoids (quercetin, luteolin, kaempferol) and phenolic content^16^ which retarded the growth of microbes. However, spoilage occurred with black and greenish patches of fungi on pizza base after 7th day onward then samples were discarded.

**Table 4:** Microbiological study of pizza base variants fortified with OSP during storage.

Storage days	Ambient condition (28 ± 2 ℃)		Refrigerated condition (5 ± 2 ℃)	
	Bacterial count (cfu/g)	Fungal count (cfu/g)	Bacterial count (cfu/g)	Fungal count (cfu/g)
**Day 1**				
Control	NG	NG	NG	NG
OSP 2%	NG	NG	NG	NG
OSP 3.5%	NG	NG	NG	NG
OSP 5%	NG	NG	NG	NG
**Day 3**				
Control	1.5 × 10^4^	2.5 × 10^4^	NG	NG
OSP 2%	1.0 × 10^4^	1.8 × 10^3^	NG	NG
OSP 3.5%	1.1 × 10^3^	2.8 × 10^3^	NG	NG
OSP 5%	0.8 × 10^3^	3.0 × 10^3^	NG	NG
**Day 5**				
Control	3.2 × 10^5^	4.8 × 10^4^	1.9 × 10^2^	1.8 × 10^3^
OSP 2%	2.2 × 10^4^	3.8 × 10^4^	0.8 × 10^2^	1.6 × 10^3^
OSP 3.5%	2.8 × 10^3^	5.1 × 10^4^	1.2 × 10^2^	2.5 × 10^2^
OSP 5%	1.9 × 10^3^	6.4 × 10^4^	0.7 × 10^2^	2.9 × 10^2^
**Day 7**				
Control	6.7 × 10^5^	9.2 × 10^5^	2.6 × 10^3^	3.2 × 10^3^
OSP 2%	4.5 × 10^4^	7.5 × 10^5^	1.9 × 10^3^	3.1 × 10^3^
OSP 3.5%	5.0 × 10^3^	9.7 × 10^4^	2.9 × 10^2^	4.2 × 10^4^
OSP 5%	4.2 × 10^3^	9.8 × 10^4^	2.1 × 10^2^	5.6 × 10^3^
**Day 9**				
Control	SNS	SNS	SNS	SNS
OSP 2%	SNS	SNS	3.9 × 10^4^	5.7 × 10^6^
OSP 3.5%	SNS	SNS	6.3 × 10^3^	8.1 × 10^5^
OSP 5%	SNS	SNS	4.8 × 10^3^	9.8 × 10^5^

*NG* no growth, *CFU* colony forming unit, *SNS* sample not survived.

In the case of refrigerated condition, no colony of microbes was detected on 1st and 3rd day but on 5th day of analysis, the control sample had 1.9 × 10^2^ cfu/g of bacterial counts and 1.8 × 10^3^ cfu/g of fungi counts whereas lower counts of bacteria (0.7 × 10^2^ cfu/g) and fungi (2.9 × 10^2^ cfu/g) were detected in OSP 5% variant (Table 4). The last day (9th) analysis showed the highest bacterial count in control variant (5.2 × 10^4^ cfu/g) and the highest fungal counts in OSP 5% variant (9.8 × 10^5^). After 9th day of storage, visible spoilage was detected due to fungal colonies. The shelf-life study concluded that refrigerated condition (5 ± 2 ℃, 30–32% relative humidity) is better than ambient storage condition for a higher shelf-life of the pizza base.

## Discussion

In the rheological study, storage modulus (G’) represents toughness and solid-like property of a sample which is related to the extent of cross-linking^17^. It is important to note that while performing frequency (ω) sweep (from 100 to 0.1 rad/s), the deviations occurred in the last stage of the experiments. These deviations represent a decline in dough density by evolving gas during fermentation with a decrease in viscosity^18^. An increase in fiber concentration with the addition of OSP may be the reason behind the tenacious and solid nature of supplemented doughs because a higher elevation in G’ was observed in 2%, 3.5%, and 5% dough variants when OSP was mixed into refined wheat flour. Previous findings support the present study and confirmed that vegetable powders enhance the G’ value of flour when added into a significant amount^19–21^.

In the case of color attributes, higher OSP concentration imparted darker color and higher yellowness to the pizza base. Previous studies also reported that yellowness and redness increased when onion skin was added into the wheat dough^22,23^. Zhang et al.^23^ observed lower lightness and darker color in quercetin enriched cookies. The changes in the color parameters occurred due to the dark red color of the onion skin of ‘NHRDF Red’ cultivar. Dark red color represents higher content of quercetin flavonoid and polyphenols which impart higher antioxidant activity^16^. It is also suggested that fiber concentration is responsible for the change in a* and b* parameters of color^22^. Likewise, a significant decrease (up to 16%) was obtained in lightness after the addition of quercetin (0.20%) in bread. In addition to this, the color of bread became dull and greyish with increased content of quercetin^24^.

According to the texture analysis, pizza samples with higher OSP content had higher hardness. Similarly, a study showed that onion powder enhanced the hardness of fortified baked roll from 264.1 ± 45.91 (control) to 695.4 ± 59.66 (5% powder)^21^. Moreover, Michalak‐Majewska et al.^21^ also found a higher increment in the chewiness of baked roll (103.7 ± 19.51) enriched with onion powder than control sample (447.8 ± 27.53) which agrees with the present study. In agreement to cohesiveness, Rubel et al.^2^ reported the same trend of cohesiveness in inulin enriched bread. A similar trend was obtained for hardness, springiness, cohesiveness, and resilience by Lin and Zhou^24^ when quercetin enriched bread was analyzed. The recorded increased hardness in enriched pizza base has corresponded to the diluting effect which imparts retention of gas, responsible for lower base volume^25^. The authors concluded that 5% OSP enriched base had the highest level of hardness and chewiness which also influenced the texture and sensory acceptance of the pizza base compared to 2% and 3.5% OSP variants.

OSP enrichment enhanced certain level of protein and fiber in the pizza base. The level of wheat flour substitution and OSP fiber content were the reason behind the change in moisture content of fortified pizza base and it was reported that the fiber content of OSP leads to higher moisture in the fortified product^26^. Sayed et al.^26^ found the similar trend for moisture content, crude fiber, crude ash, and total fat content in OSP enriched noodles which supports present study. In contrast to this, protein content decreased non-significantly in 6% OSP enriched noodle (9.30%) than control noodle sample (9.86%). Likewise, significantly lower content of crude protein was detected in banana flour (unripe) enriched bread (6.92%) than control bread (10.25%). However, fat, ash, fiber, and carbohydrates amount were in accordance with the study of Adebayo‐Oyetoro et al.^27^ Moreover, other studies have also observed physicochemical changes due to onion skin addition in bakery products^1,10,26^. Prokopov et al.^1^ recorded 1.82-times higher total fiber content in 2% substituted bread while 3% substituted bread had 2.6-times more fiber concentration than control. They found that the higher content of total fiber i.e. 69.73 ± 0.21 g/100 g in onion waste powder consequently enhanced the level of fiber in fortified bread^28^. Similarly, 4% and 6% addition of onion skin in dried noodles led to higher ash content 1.81% and 1.91%, respectively because of the replacement of onion skin^26^. It has been reported that fortification by vegetable powder generally elevated the level of WHC of the products. The higher fiber content and porous structure of raw materials are the key components for this rise^29^. For instance, standard wheat flour was fortified with mung flour, soy flour, and mango kernel flour in different ratios and analyzed for a comparative study of WHC. It was observed that composite flour had higher WHC (1.42 g H_2_O/g flour) than standard wheat flour (1.01 g H_2_O/g flour)^30^. Obtained data were not exactly in accordance with the present study values but showed a similar trend with fortified and control samples. OHC was increased with an increase in OSP content because of higher fiber content. It means control had the lowest (4.22 ± 0.04 g oil/g sample) while OSP 5% variant contained the highest (7.22 ± 0.12 g oil/g sample) OHC and the samples were significantly different (P < 0.05) from each other. Various studies found higher OHC of enriched flour than control sample^31,32^.

Phenols are important ingredients for antioxidant-rich foods. Various studies reported a higher level of total phenols and flavonoids when bakery products were fortified with onion skin and onion skin extract^3,10^. Quercetin and other flavonoids of onion impart the higher level of TPC and TFC in functional food^24^. In a similar way, 4-times more TPC concentration was increased in bread fortified with onion skin extract in 0.5% concentration^3^. Likewise, TPC was enhanced from 1.52 mg GAE/g DW (control) to 3.79 mg GAE/g DW (20% red onion) when baked rolls were analyzed after fortification^21^. Świeca et al.^33^ also found a higher TFC level (3-times than control) in the bread enriched with raw onion skin (4%). Moreover, highest concentration of TFC was obtained when 5% OSP was added in wheat bread^1^ which is in accordance with the present study.

Antioxidant activity is one of the important components for a functional food^1,3^. Piechowiak et al.^3^ were found 7-times higher antioxidant activity (DPPH) in the bread fortified with onion skin extract (0.5%). Prokopov et al.^1^ reported approximately 13-times higher DPPH activity and 6.2-times higher FRAP activity than control when wheat bread was fortified with industrial onion waste powder (5%). Likewise, Świeca et al.^33^ performed ABTS assay and found higher antioxidant potential i.e. 4.06 ± 0.16 (%/100 µg DW) in 4% onion skin enriched bread than the control (2.91 ± 0.11) in %/100 µg DW. Bedrníček et al.^12^ reported higher total flavonols (153 QE/g) in the bread fortified with 5% onion skin than control (83 QE/g) even after higher heat (180 ℃) treatment. This showed that the used onion skin had a richer amount of flavonols because the dough of the same bread (5%) had 545.58 QE/g level of flavonols.

As an important sensory parameter, color affects product acceptability and consumer preference^11^. Bread fortified with 4% and 5% industrial onion powder had darker color and least overall acceptability^1^. Likewise, onion baked rolls were prepared using red onion powder in 2.5%, 5%, 10%, and 20% concentration with wheat flour and roll with less content (2.5%) exhibited light color than 10% and 20% samples whereas sample with 20% onion powder had least overall acceptability which agrees with the present study^21^.

Higher load of microbes leads to early spoilage of food items therefore, it is necessary to retard microbial growth for extending the shelf-life of the products^34^. Murat et al.^35^ also conducted a storage study of bread in ambient (20 ± 2 ℃) and refrigerated conditions (4 ± 2 ℃) up to 21 days for bacterial and fungal counts. The results exhibited lower microbial load (< 2.00 log cfu/g) initially but it increased with storage time specifically in ambient condition. At the end, bread of ambient condition had higher microbial counts (8 log cfu/g) than refrigerated condition (6–2 log cfu/g) which support the present study. Inversely, Ijah et al.^36^ found higher bacterial (1.0 × 10^6^ cfu/g) and fungal counts (1.2 × 10^2^ cfu/g) in fresh sweet potato flour bread while wheat bread (control) had no microbial counts. The sweet potato starch provided a favourable environment to microbes and was found responsible for higher microbial load than the control. It is already proven that low temperature retards the metabolic activities of microbes^34^. Additionally, the antimicrobial agents i.e. quercetin, quercetin 3-β-d-glucoside, luteolin, and kaempferol of utilized OSP^16^ and packaging material (HDPE) might be the important reasons behind the lower range of microbial counts on the products.

In conclusion, the present research suggested that onion skin has the potential to replace onion topping on the pizza base and to increase antioxidants and flavonoids. It also showed the potential to inhibit microbial growth during storage to extend the shelf-life. Therefore, onion skin should be utilized in functional foods and nutraceuticals to mitigate reactive oxygen species (ROS) and to save the environment.

## Materials and methods

### Samples

Onion skins of fifteen Indian cultivars were procured from ICAR—Indian Agricultural Research Institute, New Delhi, India. Flavonoid quantification by HPLC (high performance liquid chromatograpy) and antioxidant activity of all the samples was carried out to choose the best cultivar skin among all. The skin of cultivar ‘NHRDF Red’ was reported to be best in terms of flavonoids content (168.77 ± 0.87 mg QE/g DW), phenolics (289.04 ± 1.31 mg GAE/g DW) and antioxidant activity (DPPH*) (94.17 ± 0.20%) and utilized in present work for incorporation in the pizza base^16^. Maida (refined wheat flour) and other items were purchased from Reliance Retail Ltd. (Delhi, India).

### Preparation of onion skin powder (OSP)

Onion skin was cleaned and washed with distilled water for complete removal of impurities and kept in deep freeze (Vestfrost Solutions, Denmark) at – 40 ºC for 24 h. The skin was freeze-dried using a lyophilizer (Mini Lyodel, Delvac Pumps, Chennai, India) with keeping the plate temperature at − 50 ºC with the pressure of 0.039 mbar and the drying process was continued up to 48 h. Freeze-dried skin was ground using a mixer-grinder (3053 Colt, Usha International Ltd, India) and sieved (400 microns) for the homogenous particle size of the powder. Prepared OSP was stored at − 30 ºC in airtight plastic containers for further use.

### Chemicals

Sulphuric acid, sodium hydroxide, aluminium chloride, Folin-Ciocalteu’s (FC) reagent, gallic acid, DPPH (2,2-diphenyl-1-picrylhydrazyl), TPTZ (2,4,6-tri(2-pyridyl)-s-triazine), ABTS (2,2′-azinobis-3-ethylbenzothiazoline-6-sulfonic acid), and other chemicals were procured from Himedia Laboratories (Mumbai, India).

### Preparation of pizza dough and base

**Figure 5:**
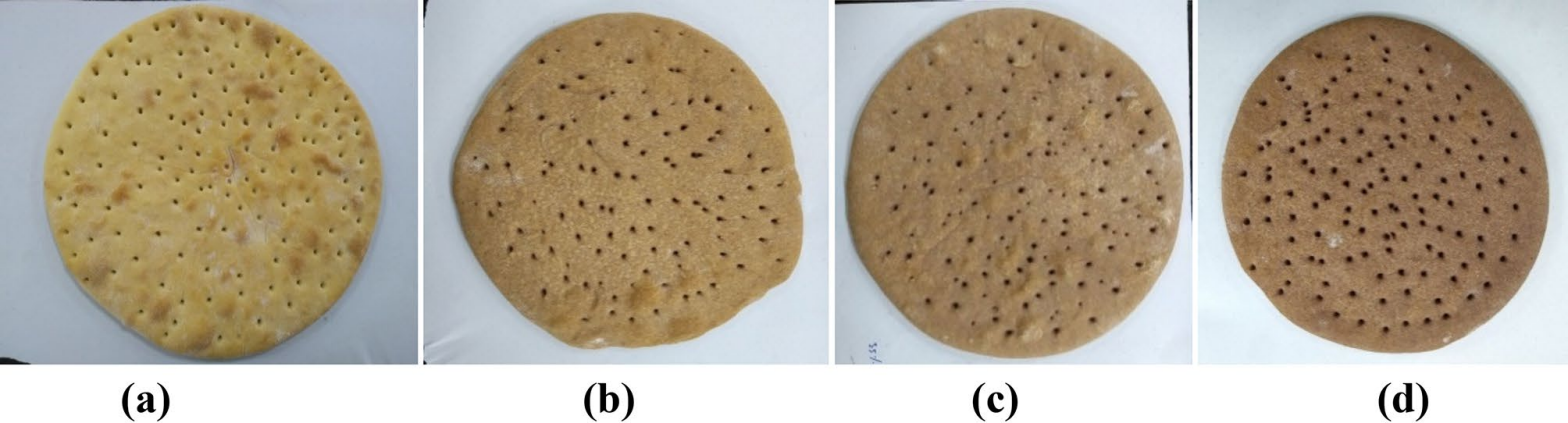
Variants of pizza base: **(a)** control, **(b)** OSP 2%, **(c)** OSP 3.5% and **(d)** OSP 5%.

Pizza dough and base were prepared using 400 g refined wheat flour (11 g/100 g protein, 0.9 g/100 g fat, 73.9 g/100 g total carbohydrates, and 0.82 g/100 g ash). The flour was replaced with OSP in 2 g/100 g, 3.5 g/100 g, and 5 g/100 g to develop three variants of pizza dough and base i.e. 2%, 3.5%, and 5% along with control (without OSP). Apart from this, 10 g sucrose, 10 mL soybean oil, 2 g sodium chloride, and 4 g instant yeast were also added to the products. The required water quantity (50 mL) as per wheat flour was added and samples were mixed thoroughly for even dough formation. Dough batches were fermented for 30 min, flattened, and put into an electric oven for 25 min at 180 ℃ to produce pizza base (Fig. 5). Pizza base variants were left to cool for 2 h at room temperature (28 ℃).

### Rheological attributes of dough

Rheological attributes were evaluated in terms of viscoelastic properties of dough samples according to Rubel et al.^2^ with slight changes using a controlled-stress rheometer (Anton Paar, Austria). Parallel plate geometry (50 mm diameter, 1 mm gap) was used to carry out the frequency (ω) sweep test. A small dough aliquot was loaded on the parallel plate (previously conditioned at 26 ℃) from the innermost part of the dough and compressed to a gap of 1 mm. The excess sample was removed and the plate rim was coated with paraffin to avoid water loss. Further, the sample was allowed to stand for 10 min at 26 ℃ for dough relaxation and thermal equilibrium. Then, an oscillatory frequency sweep test was performed (from 100 to 0.1 rad/s) of angular frequency (ω) at 0.10% strain. Loss tangent, elastic modulus (G’), and viscous modulus (G’’) were recorded through the test. Each sample was investigated in triplicate.

### Color analysis of pizza dough and base

Color analysis of prepared dough and pizza base was done using a colorimeter (Konica Minolta CR-400, Japan) as per the method of Fendri et al.^37^ Three doughs of each pizza variant were taken for evaluation, and values were measured three times. Results were shown as per the CIE L*, a*, b* scale parameter i.e. L* [lightness: 0 = black, 100 = white], a* [greenness (−), redness ( +)] and b* [blueness (−), yellowness ( +)].

### Texture analysis of pizza base

Textural properties including hardness, chewiness, cohesiveness, springiness, and resilience were investigated by the method of Rubel et al.^2^ with some changes using a texture analyzer (Stable Microsystem TA.XT, Surrey, UK) equipped with a blade set with Warner Bratzler and a 7.5 kg load cell.

### Physicochemical properties of pizza base

In the physicochemical properties, water activity was measured by water activity meter (Aqua Lab—Dewpoint, US) and moisture content was analyzed using a moisture analyzer (Citizen, India) at 28 ℃. Total fat, crude protein, ash, and total fiber content were estimated by AOAC^38^. Protein content and total fat were measured by Kjeldahl’s method and Soxhlet apparatus, respectively. Ash content was analyzed using a muffle furnace by keeping sample (3 g) for 3 h at 550 ℃ and cooled down the sample in a dryer followed by calculation. Total fiber content was measured by the calcination difference method. Carbohydrate content was estimated as per the given formulas^7^.

$${\text{Carbohydrate }}\% \, = \,{1}00 \, {-}{\text{ moisture content}}\, + \,{\text{total fiber}}\, + \,{\text{crude protein}}\, + \,{\text{total fat}}\, + \,{\text{ash content}}.$$

WHC and OHC were measured by Pourfarzad et al.^39^ was estimated by taking 3 g of sample in a pre-weighed centrifuge tube and distilled water (25 mL). The mixture was stirred and incubated for 1 h at 30 °C followed by centrifuge at 3000 × *g* for 25 min. The supernatant was removed and moisture content was measured in an oven at 50 °C for 25 min. For OHC, the sample was taken in a pre-weighed centrifuged tube and 6 mL of corn oil was added. Then, the content was thoroughly mixed and incubated for 1 h at 25 ℃ and centrifuged for 25 min at 3000 × *g*. Further, the measurement of oil density was done after discarding the supernatant. The results were expressed as g of water/oil held by g of sample on dry weight basis.

### Extract preparation and analysis of total phenols and flavonoids

Extraction was carried out by the method of Michalska et al.^40^ with minor modification. Each sample (1 g) was mixed with 10 mL of aqueous methanol (80%) and shook on a rotatory shaker at 37 ℃ on 100 × *g* for 2 h. Further, samples were centrifuged for 15 min at 4 ℃ on 12,000 × *g*. Supernatants were preserved in airtight containers at – 80 ℃ for further use.

Total phenolic content (TPC) was estimated according to Sun et al.^41^ with minor changes. Product extracts (20 μL) were mixed with 0.5 mL FC reagent (tenfold) and distilled water (5 mL) in test tubes. Test tubes were incubated for 10 min. Then, 0.5 mL of sodium carbonate (2%) was poured into each test tube and incubated for 45 min. Absorbance was taken using UV–VIS Spectrophotometer (UV-2600, Shimadzu, Kyoto, Japan) at 760 nm. Gallic acid was used as standard and the results were shown as mg gallic acid equivalents (GAE)/g DW (dry weight).

Total flavonoid content (TFC) was measured according to Benítez et al.^9^ with minor modifications. Product extracts (0.5 mL) were taken into test tubes from different samples and 1.5 mL methanol (80%) was poured into tubes. Then, potassium acetate (1 M) and aluminium chloride (10%) was poured in 0.1 mL quantity. Distilled water (2.8 mL) was poured and incubated at room temperature (28 ℃) for 30 min. The absorbance was taken via UV–VIS Spectrophotometer (UV-2600, Shimadzu, Kyoto, Japan) at 410 nm. Quercetin was used as standard and the results were expressed as mg quercetin/g DW.

### Assessment of antioxidant activity of the extracts

Antioxidant potential was determined by three different methods: ABTS^*+^ scavenging test, DPPH^*^ assay, and FRAP antioxidant assay.

ABTS^*+^ assay was performed using the method of Duan et al.^42^ with minor modifications. Potassium persulfate (2.45 mM) and ABTS (7 mM) solution were taken in 15 mL concentration each and left at room temperature for 16 h in the dark. Then, mixture dilution was done using ethanol to get 0.7–0.8 absorbance at 732 nm. This working solution (2.9 mL) was mixed with product extract (0.1 mL) and incubated for 10 min. Absorbance was determined at 732 nm and gallic acid was taken as standard. DPPH^*^ scavenging assay was carried out according to Duan et al.^42^ with some modifications. Product extract (2 mL) and 2 mL DPPH^*^ (0.4 mM) were mixed followed by incubation in dark for 1 h. Then, absorbance was measured at 517 nm via UV–VIS Spectrophotometer (UV-2600, Shimadzu, Kyoto, Japan) and gallic acid was used as standard. The percentage inhibition of ABTS^*+^ and DPPH^*^ was calculated by the given formula:

$${\text{ABTS}}^{* + } /{\text{ DPPH}}^{*} {\text{scavenging activity }}\left( \% \right) \, = \left( {{\text{A}}_{{\text{c}}} - {\text{ A}}_{{\text{s}}} } \right)/{\text{A}}_{{\text{c}}} \times { 1}00,$$

where, A_c_ = reference/control absorbance, A_s_ = sample absorbance.

FRAP antioxidant test was performed as per the method of Benzie and Strain^43^ with slight changes. Product extract (0.2 mL) was mixed with freshly prepared FRAP solution (2.8 mL) and incubated at 35 ℃ in a water bath for 15 min. The absorbance was noted at 593 by UV–VIS Spectrophotometer (UV-2600, Shimadzu, Kyoto, Japan) nm and gallic acid was taken as standard. FRAP readings were expressed as µM gallic acid/g DW.

### Sensory evaluation of fortified pizza base

Freshly prepared pizza base variants were evaluated for sensory attributes like color, symmetry, odor, flavor, mouthfeel, and overall acceptability on a nine-point hedonic scale (1 = extremely dislike, 5 = neither like nor dislike, 9 = extremely like). The sensory analysis was conducted by a panel of semi-trained personnel (male and female, n = 50). Four pieces of pizza base (one of each variant) were given to panellists randomly after coding. Potable water was provided to the panellists for palates cleansing between sample testing.

### Shelf-life study of the final products

Enriched products were undergone a storage study for shelf-life evaluation. Fresh samples were stored in two different conditions i.e. refrigerated (5 ± 2 ℃) and ambient (28 ± 2 ℃) after packaging into food grade HDPE (high-density polyethylene) bag. The shelf-life of the products was estimated by total viable counts (bacteria and yeast & mould) at a regular interval of one day. This study was carried out by the method of Ijah et al.^36^. Each sample (1 g) was taken into a test tube containing 10 mL peptone water for stock solution. One mL was taken out from each stock solution and poured into another set of test tubes having 9 mL of distilled water which made 10^–1^ (1/10) dilution. The same step was repeated to get 10^–4^ dilution (1/10,000). Then, 100 µL was spread on nutrient agar and potato dextrose agar plates for enumeration of aerobic viable bacteria and yeast & mold count, respectively. Nutrient agar plates were incubated for 24–48 h at 37 ℃ whereas potato dextrose agar plates were incubated for 3–5 days at 27 ℃ and colonies were counted as cfu/g of samples.

### Statistical analysis

All the experiments were conducted in triplicates and data was analyzed using SPSS 21.0. Data were represented as mean ± S.E.M. The difference between means was analyzed using One Way Analysis of Variance (ANOVA) test followed by Duncan’s Multiple Range Test (DMRT) and means were considered significantly different at the P < 0.05.

## Supplementary information

Supplementary Figure S1.

## References

[1] Prokopov T (2018). Effects on the quality and health-enhancing properties of industrial onion waste powder on bread. J. Food Sci. Technol..

[2] Rubel IA, Perez EE, Manrique GD, Genovese DB (2015). Fiber enrichment of wheat bread with Jerusalem artichoke inulin: Effect on dough rheology and bread quality. Food Struct..

[3] Piechowiak T, Grzelak-Błaszczyk K, Bonikowski R, Balawejder M (2020). Optimization of extraction process of antioxidant compounds from yellow onion skin and their use in functional bread production. LWT-Food Sci. Technol..

[4] Piechowiak T, Balawejder M (2019). Onion skin extract as a protective agent against oxidative stress in *Saccharomyces cerevisiae* induced by cadmium. J. Food Biochem..

[5] Gawlik-Dziki U (2015). Onion skin—Raw material for the production of supplement that enhances the health-beneficial properties of wheat bread. Food Res. Int..

[6] Sagar NA (2018). Fruit and vegetable waste: Bioactive compounds, their extraction, and possible utilization. Compr. Rev. Food Sci. Food Saf..

[7] Begum HA, Yassen T (2015). Anitmicrobial, phytochemical, ethnobotanical and proximate analysis of *Allium cepa* L. Methods.

[8] Pareek, S., Sagar, N. A., Sharma, S. & Kumar, V. Onion (*Allium cepa* L.). in *Fruit and Vegetable Phytochemicals: Chemistry and Human Health* (ed. E.M. Yahia) 1145–1162 (Wiley, New York, 2017).

[9] Benítez, V. *et al.* Characterization of industrial onion wastes (*Allium cepa* L.): Dietary fibre and bioactive compounds. *Plant Foods Hum. Nutr.* **66**(1), 48–57 (2011). 10.1007/s11130-011-0212-x 21318305

[10] Gawlik-Dziki, U. *et al.* Quality and antioxidant properties of breads enriched with dry onion (*Allium cepa* L.) skin. *Food Chem.* **138**(2–3), 1621–1628 (2013). 10.1016/j.foodchem.2012.09.151 23411290

[11] Dhen H, Rejeb IB, Boukhris H, Damergi C, Gargouri M (2018). Physicochemical and sensory properties of wheat-apricot kernels composite bread. LWT- Food Sci. Technol..

[12] Bedrníček, J. *et al.* Thermal stability and bioavailability of bioactive compounds after baking of bread enriched with different onion by-products. *Food Chem.* 126–562 (2020). 10.1016/j.foodchem.2020.126562 32155536

[13] Bernklau I (2017). Structural, textural and sensory impact of sodium reduction on long fermented pizza. Food Chem..

[14] Pagani, M. A., Lucisano, M. & Mariotti, M. Italian bakery products. in *Bakery Products Science and Technology* (eds. Zhou, W. & Hui, Y. H.) 685–721 (Wiley, New York, 2014).

[15] Gupta, C. S., Milind, Jeyarani, T. & Rajiv, J. Rheology, fatty acid profile and quality characteristics of nutrient enriched pizza base. *J. Food Sci. Technol.* **52**(5), 2926–2933 (2015). 10.1007/s13197-014-1338-2 PMC4397299 25892792

[16] Sagar NA, Pareek S, Gonzalez-Aguilar GA (2020). Quantification of flavonoids, total phenols and antioxidant properties of onion skin: A comparative study of fifteen Indian cultivars. J. Food Sci. Technol..

[17] Sánchez-Silva B, Díaz-Díaz A, Tarrío-Saavedra J, Lopez-Beceiro J, Gracia-Fernández CA, Artiaga R (2019). Thermal and rheological comparison of adhesives. J. Therm. Anal. Calorim..

[18] Dobraszczyk BJ, Morgenstern MP (2003). Rheology and the bread making process. J. Cereal Sci..

[19] Moreira R, Chenlo F, Torres MD (2013). Rheology of gluten-free doughs from blends of chestnut and rice flours. Food Bioprocess Tech..

[20] Yuan B (2017). Enrichment of bread with nutraceutical-rich mushrooms: Impact of *Auricularia auricula* (mushroom) flour upon quality attributes of wheat dough and bread. J. Food Sci..

[21] Michalak‐Majewska, M., Sołowiej, B. & Slawińska, A. Antioxidant activity, technological and rheological properties of baked rolls containing dried onions (*Allium cepa* L.). *J. Food Process. Pres. 41*(1), e12914 (2017).

[22] Gómez M (2003). Effect of dietary fibre on dough rheology and bread quality. Eur. Food Res. Technol..

[23] Zhang X, Chen F, Wang M (2014). Antioxidant and antiglycation activity of selected dietary polyphenols in a cookie model. J. Agric. Food Chem..

[24] Lin J, Zhou W (2018). Role of quercetin in the physicochemical properties, antioxidant and antiglycation activities of bread. J. Funct. Foods.

[25] Morris C, Morris GA (2012). The effect of inulin and fructooligosaccharide supplementation on the textural, rheological and sensory properties of bread and their role in weight management: A review. Food Chem..

[26] Sayed HS, Hassan NMM, El MHA (2014). The effect of using onion skin powder as a source of dietary fiber and antioxidants on properties of dried and fried noodles. Curr. Sci. J..

[27] Adebayo-Oyetoro AO, Ogundipe OO, Adeeko KN (2016). Quality assessment and consumer acceptability of bread from wheat and fermented banana flour. Food Sci. Nutr..

[28] Prokopov T (2018). Study of onion processing waste powder for potential use in food sector. Acta Aliment..

[29] Khoozani AA, Kebede B, Bekhit AEDA (2020). Rheological, textural and structural changes in dough and bread partially substituted with whole green banana flour. LWT- Food Sci. Technol..

[30] Menon L, Majumdar SD, Ravi U (2015). Development and analysis of composite flour bread. J. Food Sci. Technol..

[31] Vergara-Valencia N (2007). Fibre concentrate from mango fruit: Characterization, associated antioxidant capacity and application as a bakery product ingredient. LWT-Food Sci. Technol..

[32] Haber M, Mishyna M, Martinez JI, Benjamin O (2019). The influence of grasshopper (*Schistocerca gregaria*) powder enrichment on bread nutritional and sensorial properties. LWT Food Sci. Technol..

[33] Świeca M (2013). The influence of protein–flavonoid interactions on protein digestibility in vitro and the antioxidant quality of breads enriched with onion skin. Food Chem..

[34] Rosell CM, Gómez M (2007). Frozen dough and partially baked bread: An update. Food Rev. Int..

[35] Murat Karaoglu, M., Gürbüz Kotancilar, H. & Gurses, M. Microbiological characteristics of part-baked white pan bread during storage. *Int. J. Food Prop.* **8**(2), 355–365 (2005).

[36] Ijah UJJ, Auta HS, Aduloju MO, Aransiola SA (2014). Microbiological, nutritional, and sensory quality of bread produced from wheat and potato flour blends. Int. J. Food Sci..

[37] Fendri LB (2016). Wheat bread enrichment by pea and broad bean pods fibers: Effect on dough rheology and bread quality. LWT- Food Sci. Technol..

[38] AOAC. Official methods of analysis. in *Arlington: Association of Official Analytical Chemists* (edition 20th) (AOAC, Washington DC, 2016).

[39] Pourfarzad A, Mahdavian-Mehr H, Sedaghat N (2013). Coffee silver skin as a source of dietary fiber in bread-making: optimization of chemical treatment using response surface methodology. LWT-Food Sci. Technol..

[40] Michalska A, Amigo-Benavent M, Zielinski H, Del-Castillo MD (2008). Effect of bread making on formation of Maillard reaction products contributing to the overall antioxidant activity of rye bread. J. Cereal Sci..

[41] Sun T, Powers JR, Tang J (2007). Evaluation of the antioxidant activity of asparagus, broccoli and their juices. Food Chem..

[42] Duan, Y. *et al.* Analysis of total phenol, flavonoid content and antioxidant activity of various extraction solvents extracts from onion (*Allium cepa* L.) peels. *J. Korean Oil Chem. Soc.* **32**(3), 418–426 (2015).

[43] Benzie IF, Strain JJ (1996). The ferric reducing ability of plasma (FRAP) as a measure of “antioxidant power”: The FRAP assay. Anal. Biochem..

