# Supplementary figures and images for: Dough rheology, antioxidants, textural, physicochemical characteristics, and sensory quality of pizza base enriched with onion (Allium cepa L.) skin powder
Source: Sci Rep. 2020 Oct 29;10:18669. doi: 10.1038/s41598-020-75793-0 (PMC7596091; doi:10.1038/s41598-020-75793-0)

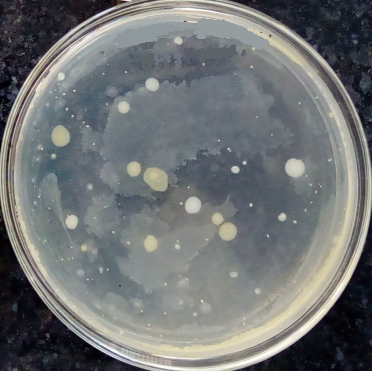

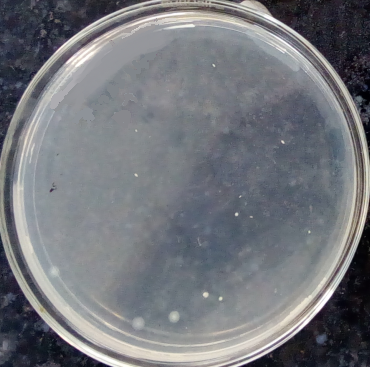


(A) (B)

**Fig. S1.** Total viable count of the samples (ambient condition: 28±2 ℃; 10-4dilution) on day 3: A. Control, B. 3.5 % OSP

Supplement: Supplementary file 1 — Supplementary Figure S1. [file 41598_2020_75793_MOESM1_ESM.docx]
